# Mosaic Subventricular Origins of Forebrain Oligodendrogenesis

**DOI:** 10.3389/fnins.2016.00107

**Published:** 2016-03-24

**Authors:** Kasum Azim, Benedikt Berninger, Olivier Raineteau

**Affiliations:** ^1^Focus Translational Neuroscience, Institute of Physiological Chemistry, University of MainzMainz, Germany; ^2^Inserm U1208, Stem Cell and Brain Research Institute, Université Lyon 1Bron, France

**Keywords:** subventricular, neural stem cell, oligodendrocyte precursor, oligodendrocyte, oligodendrogenesis, transcription factors, Wnt signaling, dorsal subventricular zone

## Abstract

In the perinatal as well as the adult CNS, the subventricular zone (SVZ) of the forebrain is the largest and most active source of neural stem cells (NSCs) that generates neurons and oligodendrocytes (OLs), the myelin forming cells of the CNS. Recent advances in the field are beginning to shed light regarding SVZ heterogeneity, with the existence of spatially segregated microdomains that are intrinsically biased to generate phenotypically distinct neuronal populations. Although most research has focused on this regionalization in the context of neurogenesis, newer findings underline that this also applies for the genesis of OLs under the control of specific patterning molecules. In this mini review, we discuss the origins as well as the mechanisms that induce and maintain SVZ regionalization. These come in the flavor of specific signaling ligands and subsequent initiation of transcriptional networks that provide a basis for subdividing the SVZ into distinct lineage-specific microdomains. We further emphasize canonical Wnts and FGF2 as essential signaling pathways for the regional genesis of OL progenitors from NSCs of the dorsal SVZ. This aspect of NSC biology, which has so far received little attention, may unveil new avenues for appropriately recruiting NSCs in demyelinating diseases.

## Introduction

Adult CNS white matter consists largely of axons, astrocytes, NG2 glia, and OLs, that are all generated in sequential steps during late development. In the rodent forebrain, 3–4 weeks after birth oligodendrocytes (OLs) develop myelin sheaths (Hartman et al., 1982; Rowitch and Kriegstein, 2010) that will wrap around axons enabling insulation and saltatory conductance of action potentials traveling down axons (reviewed in Pfeiffer et al., 1993). Major advances in the field underline that neurogenesis but also gliogenesis persists lifelong in specific germinal niches (Capilla-Gonzalez et al., 2015). The major reservoir containing neural stem cells (NSCs) in the postnatal forebrain is the subventricular zone (SVZ also referred as ventricular SVZ or subependymal zone, SEZ) of the lateral ventricle (Quinones-Hinojosa et al., 2006; Fiorelli et al., 2015). Within this germinal niche, NSCs throughout life generate new neuronal and glial cells to replenish or expand onto preexisting cell populations (Imayoshi et al., 2008; Young et al., 2013). Several recent reviews have detailed adult neurogenesis thoroughly, but much is still to be learned regarding region specific properties of NSCs in generating various subtypes of glial cells. Here, novel findings obtained are discussed to raise awareness of the importance in studying the origin of OLs in the postnatal forebrain in order to shed light onto the mechanisms that regulate their specification from spatially segregated NSCs subpopulations.

## Origins of postnatal SVZ regionalization

Immediately after birth, the SVZ undergoes major structural changes with radial glial cells [(RGCs), an embryonic form of NSCs] transforming into NSCs (Merkle et al., 2004; Tong and Alvarez-Buylla, 2014). Another subcategory of glia, ependymal glia, are generated by RGCs earlier during development (mostly between embryonic day 14 and 16, Spassky et al., 2005) and gradually mature following a caudo-rostral gradient around the lateral ventricle. NSCs, located in the SVZ during postnatal development and into adulthood are also termed as Type B1 cells (Doetsch et al., 1997) and give rise to transiently amplifying progenitors (TAPs). This latter progenitor type is identifiable by expression of Ascl1, an essential TF for the genesis of OPs from NSCs, and by short-term BrdU or EdU labeling regimes (Parras et al., 2004; Nakatani et al., 2013). Noticeable cytoarchitectural and transcriptional differences are observed between the different microdomains of the SVZ that are believe to dictate the timing and genesis of neuronal lineages (reviewed in Weinandy et al., 2011; Fiorelli et al., 2015) as well as astrocyte lineages (reviewed in Tabata, 2015). It is now evident that the diversity of neural subtypes generated after birth is larger than first believed (Merkle et al., 2007, 2014; Fiorelli et al., 2015), and emerging evidences suggest that this is now also apparent for the subtypes of glial cells.

Recent lineage tracing studies have revealed that embryonic day 10.5 NSCs (i.e., RGCs) generate all 3 major lineages, i.e., neuronal, astrocytic and oligodendroglial populations (Eckler et al., 2015). Thus, although the existence of lineage restricted RGCs clones has previously been suggested (Franco et al., 2012), it appears that multipotent NSCs prevail during early embryonic forebrain development. It is currently unknown if NSCs clones capable of giving rise to all 3 lineages are evident later in adulthood. Indeed, recent transcriptional and *in vitro* evidences suggest that segregated clones of lineage specific NSCs are observed in adulthood (Ortega et al., 2013; Llorens-Bobadilla et al., 2015), implying that adult NSCs may behave as restricted progenitors. Throughout postnatal life, the diversity in the genesis of different neural cell types is further complexed by their spatiotemporal origin within the SVZ, contrasting with previous beliefs of the SVZ as a reservoir containing a homogeneous NSC population. The events that drive genesis of OLs in a region-dependent manner within the SVZ is the focus of the present review.

Several studies have stressed regional differences in the embryonic origin and neural subtype generation from postnatal and adult SVZ-NSCs. Fate mapping approaches using Cre recombinase under the control of pallial and subpallial transcription factor (TF) promoters have collectively identified that SVZ microdomains are derived from their embryonic counterparts. For example, the medial ganglionic eminence, the lateral ganglionic eminence, and the embryonic cortex generate NSCs that populate the medial (i.e., septal), lateral (i.e., striatal), and dorsal (i.e., cortical) aspects of the adult SVZ, respectively (Ventura and Goldman, 2007; Young et al., 2007). These initial studies identified panels of key embryonic pallial regulators (Emx1, Pax6, Tbr2, Tbr1, Neurog2) whose expression is restricted to the dorsal most regions of the postnatal and adult SVZ. Subpallial markers (Dlx1/2/5, Gsh1/2, Ascl1, Nkx2.1, Nkx6.2) and septal markers (Zic1/3) are expressed more ventrally in the lateral and medial regions of the SVZ, respectively (Kohwi et al., 2007; Young et al., 2007; Batista-Brito et al., 2008; Winpenny et al., 2011; Azim et al., 2012a; Merkle et al., 2014; Sequerra, 2014). This implies that regionally segregated NSCs are primed and regulated in a timely manner for the generation of neural cells subtypes and suggests that intrinsic mechanisms coupled to environmental cues (see below) are major rate determinants of NSC fates in generating both neuronal and glial cells. In addition, recent retroviral barcode labeling of embryonic NSCs (or RGCs) have demonstrated the absence of direct linear relationship of adult or postnatal NSCs from their embryonic counterparts. Thus, the roots of postnatal and adult NSCs are apparently derived from subset of quiescent, segregated and clonally distinct embryonic progenitors from around E11.5 (Fuentealba et al., 2015). These specialized NSCs form by segregation into quiescent NSCs during embryonic development and retain their positional information onto different subregions of the postnatal SVZ through to adulthood, likely in the form of TFs.

**Figure 1:**
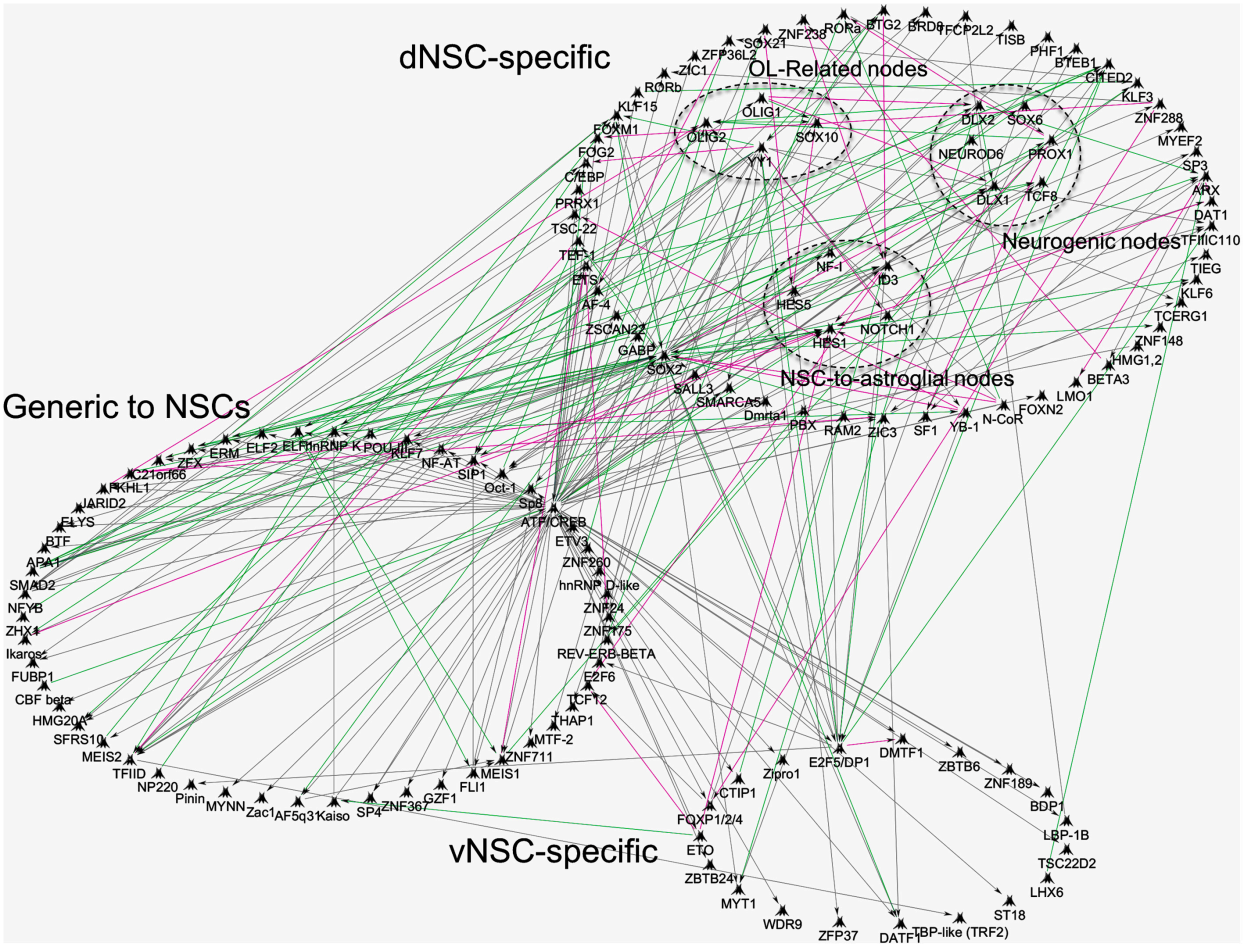
**Transcriptional network interactions in regionalized postnatal NSCs of the postnatal subventricular zone**. Previously published datasets of genes enriched in postnatal: (1) dNSCs compared to E14 dorsal RGCs and Adult NSCs; (2) vNSCs compared to E14 ventral RGCs and Adult NSCs, were re-analyzed and processed onto Genego Metacore “Direct Interactions” algorithm coupled to Dijkstra's shortest path interface to connect genes based on literature evidences. TFs were focused for analysis and grouped into categories that show dNSC, vNSC or generic enrichment (i.e., common to both). Some nodes were grouped together for illustrative purposes, for example, TFs specific to oligodendrogenesis. Green, magenta and gray lines mark activation, inhibition or unspecified interaction respectively, in TF function or direct gene regulation.

Recently, the whole transcriptome of isolated region specific postnatal NSCs has been resolved and offers new avenues to pursue in-depth analyses of SVZ regionalization (Azim et al., 2015). This study identified transcriptional differences between region specific NSCs by means of TF expression (Azim et al., 2015), that could be dependent on environmental cues, some of which are discussed below (reviewed further in Tong and Alvarez-Buylla, 2014; Fiorelli et al., 2015). Additional network interaction analysis was performed on our recently published datasets, confirming many of the above described TFs, whose expression is enriched within specific postnatal SVZ microdomains (Supplementary Tables 8, 9, Azim et al., 2015). The numbers of generic and regionally enriched TFs in postnatal NSCs compared to embryonic or adult NSCs are illustrated in Figure 1. It is noticeable that transcriptional cues regulating the switch in glial subtype specification and TFs essential for oligodendrogenesis (e.g., Olig1/2) are abundantly expressed in isolated postnatal dorsal NSCs (dNSCs) (Fuentealba et al., 2015) (see Figure 1 below) and are associated with the expression of more generic TFs, such as Ascl1 also known to be essential for oligodendrogenesis (Nakatani et al., 2013). These analyses underline the vast extent of TF complexity, which is prevalent in dNSCs compared to their ventral NSC (vNSC) counterparts, and which is likely to be causative for the greater diversities of neural lineages generated from the dorsal SVZ. Furthermore, these findings imply that the action or “networks” of multiple TFs are prerequisites in generating the large diversity of neural lineages observed during postnatal life. Future studies will identify such TF networks and foster further analyses in their relation to the timely genesis of defined neural lineages.

## Regionalized germinal origin of oligodendrocytes

Original studies of oligodendrogenesis had assumed that all SVZ-NSCs (RGCs) during embryonic development contribute and specify oligodendrocytes progenitors (OPs), based on cells of the oligodendroglial lineage being detected in all regions of the forebrain (reviewed in Richardson et al., 2006, see also Rubenstein and Rakic, 2013 for a comprehensive overview of OP migration in the forebrain). Most oligodendrogenesis studies to date assume the SVZ after birth to be a single homogeneous germinal zone. As a consequence, researchers in this field generally do not subdivide the SVZ into distinct microdomains for assessing their contribution to OL generation. Because of clear regional differences, this might result in underestimations or inconsistencies in reported findings. For example, Menn et al. (2006). described convincingly that approximately 1/20 of all newly generated cells from adult SVZ-NSCs generate OLs, but also present evidences that this ratio varies considerably depending of rostro-caudal coordinates. Other cre-lox transgenic approaches provide additional information on the origin of OL that are retained into adulthood by demonstrating that they are derived from Emx1+ dNSCs during early postnatal life (Kessaris et al., 2006). This dorsal origin of OLs at postnatal and adult stages contrast with embryonic development, when cohorts of OPs are generated from ventral or lateral forebrain sources, at E12.5 and E15.5 respectively. These OPs are eventually eliminated, presumably due to lack of appropriate survival factors (Richardson et al., 2006). Thus, the final surge of highly migratory dNSC-derived OPs ultimately fulfills its purpose in mediating forebrain myelination (Kessaris et al., 2006). It remains to be determined if postnatal dNSCs of the Emx1 lineage are intrinsically primed in generating new OL lineage cells as well as the role of dorsally enriched environmental cues in triggering OLs migration and maturation after birth. The identity of these signals may be similar to those acting earlier during development, as suggested by the dorsal enrichment of some TGFβ family members (e.g., BMP4) in the postnatal SVZ that have been described to drive OP migration into the cortex during embryonic development (Choe et al., 2014). Understanding the mechanisms that regulate oligodendrogenesis from a default origin and/or lineage restricted NSCs clones (Ortega et al., 2013; Llorens-Bobadilla et al., 2015) represents an essential first step for translational strategies aimed at stimulating endogenous forebrain NSCs.

## Extrinsic regulation of oligodendrocyte specification

**Figure 2:**
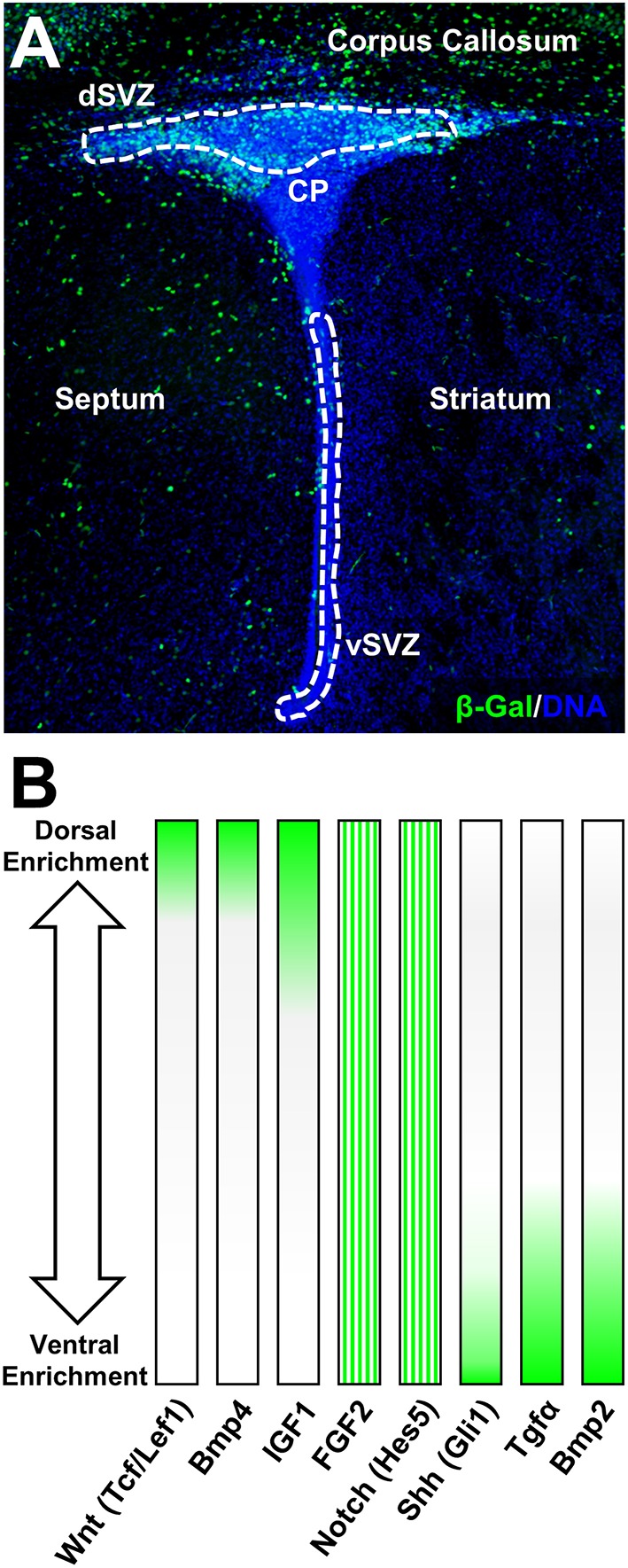
**Canonical Wnt-signaling demarcates dorsal forebrain germinal microdomains**. **(A)** Overview of the β-Gal (β-galactosidase) intensity in canonical Wnt-report mouse line highlighting exclusive expression in the dSVZ and in the adjacent corpus callosum at postnatal day 4. For staining procedures (see Azim et al., 2014a). **(B)** Examples of pathway-specific target genes and signaling ligand expression gradients according to previous published studies (Azim et al., 2012, 2014a, 2015). FGF2 and Notch (Hes5) are homogeneously distributed in the P4 SVZ and gradients illustrate relative expression intensities based on previous qPCR validation.

During postnatal life, signaling ligands are expressed by multiple sources and regulate NSC behaviors in both autocrine and paracrine manners. Expression of these ligands is observed in the various cell types forming the niche, which they also reach by the vasculature (Tavazoie et al., 2008), or more distance sources such as the choroid plexus through the cerebral spinal fluid (Falcao et al., 2012). During postnatal development and to some extent into adulthood, several generic ligands, i.e., Notch ligands, FGFs, EGF, chemokines, members of the BMP family are detected (Johe et al., 1996; Tanigaki et al., 2001; Fiorelli et al., 2015; Grinspan, 2015), and influence NSCs maintenance (see Figure 2, reviewed elsewhere in broader SVZ-oligodendrogenesis contexts, El Waly et al., 2014; Capilla-Gonzalez et al., 2015). Other ligands show regional enrichment and participate in the regionalization of the postnatal SVZ. For example, ventrally secreted Shh, which act in concert with Fgf8 during embryonic development, initiates expression of TFs of the Gsh and Nkx families as inducers of the early medial (MGE (Nkx2.1+) and lateral ventricular zones [LGE (Gsh2+) (Cocas et al., 2009]. Noticeably, Shh expression persists into adulthood to maintain SVZ regionalization (Palma et al., 2005; Ihrie et al., 2011). Those enriched in cells of the postnatal dSVZ comprise IGF1, Bmp4, Bmp7, and potent canonical Wnt-ligands such as Rspo1,2, that have long been described to dorsalize the forebrain during development (Takahashi and Liu, 2006; Bond et al., 2012; Harrison-Uy and Pleasure, 2012; Choe et al., 2014; Azim et al., 2015; see Figure 2). Importantly, receptors of some distantly secreted patterning ligands are also showing preferential regional expression. For example, this is the case for FGFR1 and FGFR2 which show dorsal enrichment in the postnatal SVZ (Azim et al., 2012) and may therefore regionally integrate FGF2 signaling in promoting NP/OP specification, proliferation and migration (Garcia-Gonzalez et al., 2010; Murcia-Belmonte et al., 2014). Thus, local expression of morphogens combined with regional expression of receptors or downstream effectors of distantly secreted ones are likely to act together in initiating TF expression that stimulates and maintains microdomain heterogeneity during postnatal life. In the case of dorsalizing ligands that promote oligodendrogenesis, Wnt-signaling appears to be a central candidate onto which other signaling pathways converge (see below). Refer to Guo et al (Guo et al., 2015) for a recent comprehensive review of Wnt signaling in the distinct stages of OL differentiation and CNS regions.

The initial dorsalizing trigger of the forebrain and subsequent inducer of oligodendrogenesis at birth, are derived from the choroid plexus which releases several canonical Wnt-ligands (Harrison-Uy and Pleasure, 2012; Azim et al., 2014a). In turn, newly generated OL lineage cells provide further added autocrine support by secreting Wnt3 and possibly other canonical Wnt ligands (Harrison-Uy and Pleasure, 2012; Ortega et al., 2013; Azim et al., 2014a). In the postnatal SVZ, active Wnt-signaling as per the Wnt-reporter Bat-gal transgenic exhibits intense signal that is detected in dorsal NSCs and TAPs as well as within OL lineage cells in the overlying corpus callosum, which are still maturing at this stage (Figure 2; Azim et al., 2014a). Canonical Wnt activation by either genetic or pharmacological means promotes the generation of OPs by dNSCs (Azim et al., 2014a,b). Wnt-signaling however appears to be an additive mechanism in enhancing the genesis of OL lineage cells. Indeed, ablation in the transcriptional activity of β-catenin does not alter the numbers of newly specified dNSC-derived OPs (Azim et al., 2014a). This is likely to be due to the presence of other Wnt effectors that positively regulates oligodendrocyte differentiation in a manner independent of Wnt/β-catenin signaling, i.e., Tcf7l2 (Hammond et al., 2015), (highly enriched in expression in dNSCs compared to vNSCs, Azim et al., 2015), as well as in the activity of other signaling pathways such as FGF2 (Azim et al., 2014b). Further studies are needed to address the mechanisms by which Tcf7l2 drives NSC-to-OP fates in this context, as well as the involvement of other signaling ligands in regulating this process. Interestingly, enforcing genetically downstream transcription of Wnt-signaling in vNSCs immediately after birth does not alter the numbers of newly generated OPs from adjacent NSC sources (Azim et al., 2014a), while pharmacological activation of Wnt-signaling and infusion of FGF2 only partly induces vNSC-to-OP specification (Azim et al., 2012, 2014b). Notably, even at embryonic stages when the developing forebrain is relatively more plastic, ectopic activation of downstream Wnt-signaling in vSVZ regions only partially promotes dorsalization, although few ventral markers, Nkx2.1, Gsh2, and Ascl1 are down-regulated (Backman et al., 2005). Altogether, these observations suggest the early appearance of epigenetic barriers, multiple inhibitory factors and lack of intrinsic TF networks permitting oligodendrogenesis in more ventral SVZ microdomains. Thus, signaling molecules such as Wnts together with FGF2 act in concert as major inducers of dorsally derived oligodendrogenesis during postnatal development and adulthood.

## Cross-talk of signaling ligands in regulating downstream Wnt-signaling

In the postnatal forebrain, immediately following birth, relatively few specific lineage directive cues that boost the genesis of OPs from dNSCs have been identified. These, appear to ultimately converge onto activation of common TFs that are considered acting downstream of the Wnt-signaling machinery. As active Wnt canonical signaling is profusely detected in the dSVZ and absent in other SVZ microdomains (Figure 2A), few ligands have been identified that have the capacity to directly regulate dorsalization through β-catenin nuclear accumulation. These include, FGF2 as well as BMP4 or EGF that respectfully positively or negatively regulate β-catenin nuclear function, implying multiple modes of regulation by signaling ligands known to be present in the dorsal SVZ (Azim et al., 2014b). In this respect, high concentrations of FGF2 is one of few triggers able to induce some aspects of dorsal identity and oligodendrogenesis in the postnatal SVZ (Naruse et al., 2006). This is likely to occur, at least in part, by inhibition of GSK3β (Azim et al., 2014b), presumably via activation of FGFR1 and FGFR2 that are enriched in the dSVZ (Azim et al., 2012). The precise signaling machineries acting downstream of FGFRs and involved in this cross-talk are unknown since multiple developmentally relevant kinases are able to phosphorylate and therefore inhibit GSK3β (Grimes and Jope, 2001). At later differentiation stages *in vitro*, other ligands such as IGF1 or PDGF upregulate major myelin-related genes via β-catenin activity, dependent of GSK3β signaling (Ye et al., 2010; Chew et al., 2011). This suggests the existence of a cross-talk between multiple signaling pathways and the canonical Wnt pathway, possibly converging onto the inhibition of GSK3β. This kinase is involved in several cellular processes and is generally considered as a negative regulator in neurodevelopmental contexts. Its expression in postnatal NSCs is considerably higher compared to others cells in the SVZ (Azim et al., 2015), and developmentally it is often associated with regulation of the Wnt-signaling pathway, with lesser weight on other developmentally important pathways such as Notch, Shh, etc. (reviewed in Kim and Snider, 2011). This was recently confirmed within the SVZ microdomains, in which pharmacological inhibition of GSK3β induces the expression of Wnt target genes by multiple folds in parallel to oligodendrogenesis, whereas target genes specific to other pathways (i.e., Notch, Shh, Bmps) are either very subtly affected or are unaltered (Azim et al., 2014b). Additionally, GSK3β further regulates later stages of OL differentiation in parallel to other signaling pathways (Azim and Butt, 2011; Meffre et al., 2015). In this respect, it is noteworthy mentioning FGF2 activation of Erk1/2 signaling through FGFR1/2, and its cross talk with Akt/mTor signaling in regulating OL migration (Ishii et al., 2014; Murcia-Belmonte et al., 2015), differentiation and survival (Guardiola-Diaz et al., 2012; Dai et al., 2014). Further studies in the field are required to address the concerted role of “generic” (i.e., EGF, VEGFs, HGFs, etc.) and regionaly acting (i.e., FGF2, Wnt) signaling ligands in mediating these effects via the induction of specific TF networks (see Figure 1).

## Summary and future outlook

In this review, the known mechanisms essential for inducing oligodendrogenesis have been discussed that altogether underline a strict spatial coding within segregated NSC populations of the postnatal dSVZ. Evidences for the existence of lineage specific microdomains in primates (Azim et al., 2013), coupled to the demonstrated origin of OLs from dorsal RGCs in developing human brain (Rakic and Zecevic, 2003), and activation of the SVZ in human Multiple Sclerosis lesions (Nait-Oumesmar et al., 2007), emphasizes that the SVZ should be sampled in 3D for recruitment of region-specific NSCs. Ultimately, identifying mechanisms that regulate oligodendrogenesis from specific subsets of NSCs, will serve as a starting basis for future translational studies.

## Author contributions

KA: Main contributing author and wrote the article; BB and OR: Financial support, manuscript editing.

### Conflict of interest statement

The authors declare that the research was conducted in the absence of any commercial or financial relationships that could be construed as a potential conflict of interest. The reviewer FD and handling Editor declared their shared affiliation, and the handling Editor states that the process nevertheless met the standards of a fair and objective review.

## Abbreviations

NSC: neural stem cells
OLs: oligodendrocytes
OPs: oligodendrocyte progenitors
SVZ: subventricular
dSVZ: dorsal subventricular zone
dNSCs: dorsal neural stem cells
vNSCs: ventral neural stem cells
RGCs: radial glial cells
vSVZ: ventral subventricular zone.

## References

[B1] Azim K. Butt A. M. (2011). GSK3beta negatively regulates oligodendrocyte differentiation and myelination *in vivo*. Glia 59, 540–553. 10.1002/glia.21122 21319221

[B2] Azim K. Fiorelli R. Zweifel S. Hurtado-Chong A. Yoshikawa K. Slomianka L.. (2012a). 3-dimensional examination of the adult mouse subventricular zone reveals lineage-specific microdomains. PLoS ONE 7:e49087. 10.1371/journal.pone.0049087 23166605 PMC3499551

[B3] Azim K. Fischer B. Hurtado-Chong A. Draganova K. Cantu C. Zemke M.. (2014a). Persistent Wnt/beta-catenin signaling determines dorsalization of the postnatal subventricular zone and neural stem cell specification into oligodendrocytes and glutamatergic neurons. Stem Cells 32, 1301–1312. 10.1002/stem.1639 24449255

[B4] Azim K. Hurtado-Chong A. Fischer B. Kumar N. Zweifel S. Taylor V.. (2015). Transcriptional hallmarks of heterogeneous neural stem cell niches of the subventricular zone. Stem Cells 33, 2232–2242. 10.1002/stem.2017 25827345

[B5] Azim K. Raineteau O. Butt A. M. (2012). bIntraventricular injection of FGF-2 promotes generation of oligodendrocyte-lineage cells in the postnatal and adult forebrain. Glia 60, 1977–1990. 10.1002/glia.22413 22951928

[B6] Azim K. Rivera A. Raineteau O. Butt A. M. (2014b). GSK3beta regulates oligodendrogenesis in the dorsal microdomain of the subventricular zone via Wnt-beta-catenin signaling. Glia 62, 778–779. 10.1002/glia.22641 24677550

[B7] Azim K. Zweifel S. Klaus F. Yoshikawa K. Amrein I. Raineteau O. (2013). Early decline in progenitor diversity in the marmoset lateral ventricle. Cereb. Cortex 23, 922–931. 10.1093/cercor/bhs085 22473896

[B8] Backman M. Machon O. Mygland L. van den Bout C. J. Zhong W. Taketo M. M.. (2005). Effects of canonical Wnt signaling on dorso-ventral specification of the mouse telencephalon. Dev. Biol. 279, 155–168. 10.1016/j.ydbio.2004.12.010 15708565

[B9] Batista-Brito R. Close J. Machold R. Fishell G. (2008). The distinct temporal origins of olfactory bulb interneuron subtypes. J. Neurosci. 28, 3966–3975. 10.1523/JNEUROSCI.5625-07.2008 18400896 PMC2505353

[B10] Bond A. M. Bhalala O. G. Kessler J. A. (2012). The dynamic role of bone morphogenetic proteins in neural stem cell fate and maturation. Dev. Neurobiol. 72, 1068–1084. 10.1002/dneu.22022 22489086 PMC3773925

[B11] Capilla-Gonzalez V. Herranz-Perez V. Garcia-Verdugo J. M. (2015). The aged brain: genesis and fate of residual progenitor cells in the subventricular zone. Front. Cell. Neurosci. 9:365. 10.3389/fncel.2015.00365 26441536 PMC4585225

[B12] Chew L. J. Shen W. Ming X. Senatorov V. V. Jr. Chen H. L. Cheng Y.. (2011). SRY-box containing gene 17 regulates the Wnt/beta-catenin signaling pathway in oligodendrocyte progenitor cells. J. Neurosci. 31, 13921–13935. 10.1523/JNEUROSCI.3343-11.2011 21957254 PMC3227525

[B13] Choe Y. Huynh T. Pleasure S. J. (2014). Migration of oligodendrocyte progenitor cells is controlled by transforming growth factor beta family proteins during corticogenesis. J. Neurosci. 34, 14973–14983. 10.1523/JNEUROSCI.1156-14.2014 25378163 PMC4220029

[B14] Cocas L. A. Miyoshi G. Carney R. S. Sousa V. H. Hirata T. Jones K. R.. (2009). Emx1-lineage progenitors differentially contribute to neural diversity in the striatum and amygdala. J. Neurosci. 29, 15933–15946. 10.1523/JNEUROSCI.2525-09.2009 20016109 PMC3679174

[B15] Dai J. Bercury K. K. Macklin W. B. (2014). Interaction of mTOR and Erk1/2 signaling to regulate oligodendrocyte differentiation. Glia 62, 2096–2109. 10.1002/glia.22729 25060812 PMC4406223

[B16] Doetsch F. Garcia-Verdugo J. M. Alvarez-Buylla A. (1997). Cellular composition and three-dimensional organization of the subventricular germinal zone in the adult mammalian brain. J. Neurosci. 17, 5046–5061. 9185542 10.1523/JNEUROSCI.17-13-05046.1997 PMC6573289

[B17] Eckler M. J. Nguyen T. D. McKenna W. L. Fastow B. L. Guo C. Rubenstein J. L.. (2015). Cux2-positive radial glial cells generate diverse subtypes of neocortical projection neurons and macroglia. Neuron 86, 1100–1108. 10.1016/j.neuron.2015.04.020 25996137 PMC4441766

[B18] El Waly B. Macchi M. Cayre M. Durbec P. (2014). Oligodendrogenesis in the normal and pathological central nervous system. Front. Neurosci. 8:145. 10.3389/fnins.2014.00145 24971048 PMC4054666

[B19] Falcao A. M. Marques F. Novais A. Sousa N. Palha J. A. Sousa J. C. (2012). The path from the choroid plexus to the subventricular zone: go with the flow! Front. Cell. Neurosci. 6:34. 10.3389/fncel.2012.00034 22907990 PMC3414909

[B20] Fiorelli R. Azim K. Fischer B. Raineteau O. (2015). Adding a spatial dimension to postnatal ventricular-subventricular zone neurogenesis. Development 142, 2109–2120. 10.1242/dev.119966 26081572

[B21] Franco S. J. Gil-Sanz C. Martinez-Garay I. Espinosa A. Harkins-Perry S. R. Ramos C.. (2012). Fate-restricted neural progenitors in the mammalian cerebral cortex. Science 337, 746–749. 10.1126/science.1223616 22879516 PMC4287277

[B22] Fuentealba L. C. Rompani S. B. Parraguez J. I. Obernier K. Romero R. Cepko C. L.. (2015). Embryonic origin of postnatal neural stem cells. Cell 161, 1644–1655. 10.1016/j.cell.2015.05.041 26091041 PMC4475276

[B23] Garcia-Gonzalez D. Clemente D. Coelho M. Esteban P. F. Soussi-Yanicostas N. de Castro F. (2010). Dynamic roles of FGF-2 and Anosmin-1 in the migration of neuronal precursors from the subventricular zone during pre- and postnatal development. Exp. Neurol. 222, 285–295. 10.1016/j.expneurol.2010.01.006 20083104

[B24] Grimes C. A. Jope R. S. (2001). The multifaceted roles of glycogen synthase kinase 3beta in cellular signaling. Prog. Neurobiol. 65, 391–426. 10.1016/S0301-0082(01)00011-9 11527574

[B25] Grinspan J. B. (2015). Bone morphogenetic proteins: inhibitors of myelination in development and disease. Vitam. Horm. 99, 195–222. 10.1016/bs.vh.2015.05.005 26279377

[B26] Guardiola-Diaz H. M. Ishii A. Bansal R. (2012). Erk1/2 MAPK and mTOR signaling sequentially regulates progression through distinct stages of oligodendrocyte differentiation. Glia 60, 476–486. 10.1002/glia.22281 22144101 PMC3265651

[B27] Guo F. Lang J. Sohn J. Hammond E. Chang M. Pleasure D. (2015). Canonical Wnt signaling in the oligodendroglial lineage–puzzles remain. Glia 63, 1671–1693. 10.1002/glia.22813 25782433

[B28] Hammond E. Lang J. Maeda Y. Pleasure D. Angus-Hill M. Xu J.. (2015). The Wnt effector transcription factor 7-like 2 positively regulates oligodendrocyte differentiation in a manner independent of Wnt/beta-catenin signaling. J. Neurosci. 35, 5007–5022. 10.1523/JNEUROSCI.4787-14.2015 25810530 PMC6705374

[B29] Harrison-Uy S. J. Pleasure S. J. (2012). Wnt signaling and forebrain development. Cold Spring Harb. Perspect. Biol. 4:a008094. 10.1101/cshperspect.a008094 22621768 PMC3385962

[B30] Hartman B. K. Agrawal H. C. Agrawal D. Kalmbach S. (1982). Development and maturation of central nervous system myelin: comparison of immunohistochemical localization of proteolipid protein and basic protein in myelin and oligodendrocytes. Proc. Natl. Acad. Sci. U.S.A. 79, 4217–4220. 10.1073/pnas.79.13.4217 6180437 PMC346609

[B31] Ihrie R. A. Shah J. K. Harwell C. C. Levine J. H. Guinto C. D. Lezameta M.. (2011). Persistent sonic hedgehog signaling in adult brain determines neural stem cell positional identity. Neuron 71, 250–262. 10.1016/j.neuron.2011.05.018 21791285 PMC3346180

[B32] Imayoshi I. Sakamoto M. Ohtsuka T. Takao K. Miyakawa T. Yamaguchi M.. (2008). Roles of continuous neurogenesis in the structural and functional integrity of the adult forebrain. Nat. Neurosci. 11, 1153–1161. 10.1038/nn.2185 18758458

[B33] Ishii A. Furusho M. Dupree J. L. Bansal R. (2014). Role of ERK1/2 MAPK signaling in the maintenance of myelin and axonal integrity in the adult CNS. J. Neurosci. 34, 16031–16045. 10.1523/JNEUROSCI.3360-14.2014 25429144 PMC4244469

[B34] Johe K. K. Hazel T. G. Muller T. Dugich-Djordjevic M. M. McKay R. D. (1996). Single factors direct the differentiation of stem cells from the fetal and adult central nervous system. Genes Dev. 10, 3129–3140. 10.1101/gad.10.24.3129 8985182

[B35] Kessaris N. Fogarty M. Iannarelli P. Grist M. Wegner M. Richardson W. D. (2006). Competing waves of oligodendrocytes in the forebrain and postnatal elimination of an embryonic lineage. Nat. Neurosci. 9, 173–179. 10.1038/nn1620 16388308 PMC6328015

[B36] Kim W. Y. Snider W. D. (2011). Functions of GSK-3 signaling in development of the nervous system. Front. Mol. Neurosci. 4:44. 10.3389/fnmol.2011.00044 22125510 PMC3221276

[B37] Kohwi M. Petryniak M. A. Long J. E. Ekker M. Obata K. Yanagawa Y.. (2007). A subpopulation of olfactory bulb GABAergic interneurons is derived from Emx1- and Dlx5/6-expressing progenitors. J. Neurosci. 27, 6878–6891. 10.1523/JNEUROSCI.0254-07.2007 17596436 PMC4917362

[B38] Llorens-Bobadilla E. Zhao S. Baser A. Saiz-Castro G. Zwadlo K. Martin-Villalba A. (2015). Single-cell transcriptomics reveals a population of dormant neural stem cells that become activated upon brain injury. Cell Stem Cell 17, 329–340. 10.1016/j.stem.2015.07.002 26235341

[B39] Meffre D. Massaad C. Grenier J. (2015). Lithium chloride stimulates PLP and MBP expression in oligodendrocytes via Wnt/beta-catenin and Akt/CREB pathways. Neuroscience 284, 962–971. 10.1016/j.neuroscience.2014.10.064 25451297

[B40] Menn B. Garcia-Verdugo J. M. Yaschine C. Gonzalez-Perez O. Rowitch D. Alvarez-Buylla A. (2006). Origin of oligodendrocytes in the subventricular zone of the adult brain. J. Neurosci. 26, 7907–7918. 10.1523/JNEUROSCI.1299-06.2006 16870736 PMC6674207

[B41] Merkle F. T. Fuentealba L. C. Sanders T. A. Magno L. Kessaris N. Alvarez-Buylla A. (2014). Adult neural stem cells in distinct microdomains generate previously unknown interneuron types. Nat. Neurosci. 17, 207–214. 10.1038/nn.3610 24362763 PMC4100623

[B42] Merkle F. T. Mirzadeh Z. Alvarez-Buylla A. (2007). Mosaic organization of neural stem cells in the adult brain. Science 317, 381–384. 10.1126/science.1144914 17615304

[B43] Merkle F. T. Tramontin A. D. Garcia-Verdugo J. M. Alvarez-Buylla A. (2004). Radial glia give rise to adult neural stem cells in the subventricular zone. Proc. Natl. Acad. Sci. U.S.A. 101, 17528–17532. 10.1073/pnas.0407893101 15574494 PMC536036

[B44] Murcia-Belmonte V. Esteban P. F. Martinez-Hernández J. Gruart A. Luján R. Delgado-García J. M.. (2015). Anosmin-1 over-expression regulates oligodendrocyte precursor cell proliferation, migration and myelin sheath thickness. Brain Struct. Funct. [Epub ahead of print]. 10.1007/s00429-014-0977-4 25662897

[B45] Murcia-Belmonte V. Medina-Rodriguez E. M. Bribian A. de Castro F. Esteban P. F. (2014). ERK1/2 signaling is essential for the chemoattraction exerted by human FGF2 and human anosmin-1 on newborn rat and mouse OPCs via FGFR1. Glia 62, 374–386. 10.1002/glia.22609 24375670

[B46] Nait-Oumesmar B. Picard-Riera N. Kerninon C. Decker L. Seilhean D. Hoglinger G. U.. (2007). Activation of the subventricular zone in multiple sclerosis: evidence for early glial progenitors. Proc. Natl. Acad. Sci. U.S.A. 104, 4694–4699. 10.1073/pnas.0606835104 17360586 PMC3025281

[B47] Nakatani H. Martin E. Hassani H. Clavairoly A. Maire C. L. Viadieu A.. (2013). Ascl1/Mash1 promotes brain oligodendrogenesis during myelination and remyelination. J. Neurosci. 33, 9752–9768. 10.1523/JNEUROSCI.0805-13.2013 23739972 PMC3892435

[B48] Naruse M. Nakahira E. Miyata T. Hitoshi S. Ikenaka K. Bansal R. (2006). Induction of oligodendrocyte progenitors in dorsal forebrain by intraventricular microinjection of FGF-2. Dev. Biol. 297, 262–273. 10.1016/j.ydbio.2006.05.017 16782086

[B49] Ortega F. Gascon S. Masserdotti G. Deshpande A. Simon C. Fischer J.. (2013). Oligodendrogliogenic and neurogenic adult subependymal zone neural stem cells constitute distinct lineages and exhibit differential responsiveness to Wnt signalling. Nat. Cell Biol. 15, 602–613. 10.1038/ncb2736 23644466

[B50] Palma V. Lim D. A. Dahmane N. Sanchez P. Brionne T. C. Herzberg C. D.. (2005). Sonic hedgehog controls stem cell behavior in the postnatal and adult brain. Development 132, 335–344. 10.1242/dev.01567 15604099 PMC1431583

[B51] Parras C. M. Galli R. Britz O. Soares S. Galichet C. Battiste J.. (2004). Mash1 specifies neurons and oligodendrocytes in the postnatal brain. EMBO J. 23, 4495–4505. 10.1038/sj.emboj.7600447 15496983 PMC526464

[B52] Pfeiffer S. E. Warrington A. E. Bansal R. (1993). The oligodendrocyte and its many cellular processes. Trends Cell Biol. 3, 191–197. 10.1016/0962-8924(93)90213-K 14731493

[B53] Quinones-Hinojosa A. Sanai N. Soriano-Navarro M. Gonzalez-Perez O. Mirzadeh Z. Gil-Perotin S.. (2006). Cellular composition and cytoarchitecture of the adult human subventricular zone: a niche of neural stem cells. J. Comp. Neurol. 494, 415–434. 10.1002/cne.20798 16320258

[B54] Rakic S. Zecevic N. (2003). Early oligodendrocyte progenitor cells in the human fetal telencephalon. Glia 41, 117–127. 10.1002/glia.10140 12509802

[B55] Richardson W. D. Kessaris N. Pringle N. (2006). Oligodendrocyte wars. Nat. Rev. Neurosci. 7, 11–18. 10.1038/nrn1826 16371946 PMC6328010

[B56] Rowitch D. H. Kriegstein A. R. (2010). Developmental genetics of vertebrate glial-cell specification. Nature 468, 214–222. 10.1038/nature09611 21068830

[B57] Rubenstein J. L. R. Rakic P. (2013). Comprehensive Developmental Neuroscience: Patterning and Cell Type Specification in the Developing CNS and PNS. Oxford: Academic Press.

[B58] Sequerra E. B. (2014). Subventricular zone progenitors in time and space: generating neuronal diversity. Front. Cell. Neurosci. 8:434. 10.3389/fncel.2014.00434 25565967 PMC4273657

[B59] Spassky N. Merkle F. T. Flames N. Tramontin A. D. Garcia-Verdugo J. M. Alvarez-Buylla A. (2005). Adult ependymal cells are postmitotic and are derived from radial glial cells during embryogenesis. J. Neurosci. 25, 10–18. 10.1523/JNEUROSCI.1108-04.2005 15634762 PMC6725217

[B60] Tabata H. (2015). Diverse subtypes of astrocytes and their development during corticogenesis. Front. Neurosci. 9:114. 10.3389/fnins.2015.00114 25904839 PMC4387540

[B61] Takahashi H. Liu F. C. (2006). Genetic patterning of the mammalian telencephalon by morphogenetic molecules and transcription factors. Birth Defects Res. C Embryo Today 78, 256–266. 10.1002/bdrc.20077 17061260

[B62] Tanigaki K. Nogaki F. Takahashi J. Tashiro K. Kurooka H. Honjo T. (2001). Notch1 and Notch3 instructively restrict bFGF-responsive multipotent neural progenitor cells to an astroglial fate. Neuron 29, 45–55. 10.1016/S0896-6273(01)00179-9 11182080

[B63] Tavazoie M. Van der Veken L. Silva-Vargas V. Louissaint M. Colonna L. Zaidi B.. (2008). A specialized vascular niche for adult neural stem cells. Cell Stem Cell 3, 279–288. 10.1016/j.stem.2008.07.025 18786415 PMC6864413

[B64] Tong C. K. Alvarez-Buylla A. (2014). SnapShot: adult neurogenesis in the V-SVZ. Neuron 81, 220-220.e1. 10.1016/j.neuron.2013.12.004 24411739 PMC4034739

[B65] Ventura R. E. Goldman J. E. (2007). Dorsal radial glia generate olfactory bulb interneurons in the postnatal murine brain. J. Neurosci. 27, 4297–4302. 10.1523/JNEUROSCI.0399-07.2007 17442813 PMC6672317

[B66] Weinandy F. Ninkovic J. Gotz M. (2011). Restrictions in time and space–new insights into generation of specific neuronal subtypes in the adult mammalian brain. Eur. J. Neurosci. 33, 1045–1054. 10.1111/j.1460-9568.2011.07602.x 21395847

[B67] Winpenny E. Lebel-Potter M. Fernandez M. E. Brill M. S. Gotz M. Guillemot F.. (2011). Sequential generation of olfactory bulb glutamatergic neurons by Neurog2-expressing precursor cells. Neural Dev. 6:12. 10.1186/1749-8104-6-12 21466690 PMC3087671

[B68] Ye P. Hu Q. Liu H. Yan Y. D'Ercole A. J. (2010). beta-catenin mediates insulin-like growth factor-I actions to promote cyclin D1 mRNA expression, cell proliferation and survival in oligodendroglial cultures. Glia 58, 1031–1041. 10.1002/glia.20984 20235220 PMC2917840

[B69] Young K. M. Fogarty M. Kessaris N. Richardson W. D. (2007). Subventricular zone stem cells are heterogeneous with respect to their embryonic origins and neurogenic fates in the adult olfactory bulb. J. Neurosci. 27, 8286–8296. 10.1523/JNEUROSCI.0476-07.2007 17670975 PMC6331046

[B70] Young K. M. Psachoulia K. Tripathi R. B. Dunn S. J. Cossell L. Attwell D.. (2013). Oligodendrocyte dynamics in the healthy adult CNS: evidence for myelin remodeling. Neuron 77, 873–885. 10.1016/j.neuron.2013.01.006 23473318 PMC3842597

